# How Do the Different Proteomic Strategies Cope with the Complexity of Biological Regulations in a Multi-Omic World? Critical Appraisal and Suggestions for Improvements

**DOI:** 10.3390/proteomes8030023

**Published:** 2020-09-03

**Authors:** Katrin Marcus, Thierry Rabilloud

**Affiliations:** 1 Medizinisches Proteom-Center, Medical Faculty & Medical Proteome Analysis, Center for Proteindiagnostics (PRODI) Ruhr-University Bochum Gesundheitscampus, 4 44801 Bochum, Germany; Katrin.Marcus@ruhr-uni-bochum.de; 2 Laboratory of Chemistry and Biology of Metals, Université Grenoble Alpes UMR 5249, CNRS, CEA 38054 Grenoble, France

**Keywords:** two-dimensional gel electrophoresis, proteomics, post-translational modifications, proteoforms, clinical proteomics

## Abstract

In this second decade of the 21st century, we are lucky enough to have different types of proteomic analyses at our disposal. Furthermore, other functional omics such as transcriptomics have also undergone major developments, resulting in mature tools. However, choice equals questions, and the major question is how each proteomic strategy is fit for which purpose. The aim of this opinion paper is to reposition the various proteomic strategies in the frame of what is known in terms of biological regulations in order to shed light on the power, limitations, and paths for improvement for the different proteomic setups. This should help biologists to select the best-suited proteomic strategy for their purposes in order not to be driven by raw availability or fashion arguments but rather by the best fitness for purpose. In particular, knowing the limitations of the different proteomic strategies helps in interpreting the results correctly and in devising the validation experiments that should be made downstream of the proteomic analyses.

## 1. The “City Noir” of Proteomics: Where Is the Light?

**Figure 1:**
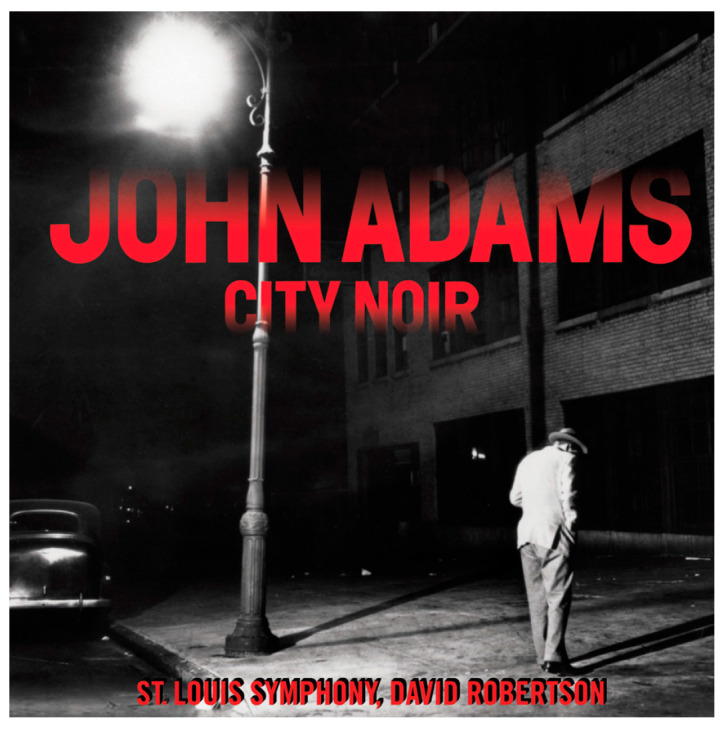
The city noir cover (reproduced with permission of Nonesuch records).

City noir is not only a wonderful piece of 20th century music composed by John Adams; it also has the wonderful cover displayed on Figure 1:

This cover is highly reminiscent of a well-known story, where you cross a person like the one on the cover, obviously looking for something under the lamppost. The following dialog ensues:

“Hey Sir, you seem to be looking for something, may I help you?”

“Sure, I’ve lost my keys”

After a search when both of you can detect nothing, you say:

“Well, I can’t see any keys, are you sure that you lost them around here?”

“Oh, probably not, but at least, here there is light…”

This story is indeed a metaphor of a well-known cognitive bias, which is called the cognitive availability bias. This bias consists of using a piece of data to make a point, even when knowing that these data are not really relevant, because the data are available, and not questioning any further the fitness of the data for the purpose. This has happened (and still happens) countless times in science and is very prevalent in proteomics and in functional omics in general. The problem is not to use a surrogate proxy for the real object of interest, biochemically speaking, but arises when the limits of the proxy are forgotten and the results over-interpreted. In order to get a fair appraisal of where the light in proteomics really is, it is first necessary to take a step back and remind us of what types of proteomics we have at our disposal and then how cells work.

## 2. The Landscape

### 2.1. The Different Proteomic Strategies

The word “proteome,” defined as the protein complement of the genome, was first used in a publication in 1995 [1]. Remarkably, this very definition tells that the proteome comes after the genome, and indeed there is no proteomic science without genomic science. Still today, when working on a new organism, a totally sequenced and well-annotated genome is a prerequisite for doing proteomics. In a sense, all proteomic data are interpreted from the genomic book. However, there are different ways to acquire and read proteomic data. Depending on the analyte that is separated and quantified, two major types of proteomics can be described.

Top-down proteomics separates and quantifies complete proteins prior to their analysis by mass spectrometry. Depending on the main tool used, top-down proteomics comes in two very different setups. 

In the first setup, two-dimensional gel electrophoresis (2DGE) is the tool used to separate and quantify proteins, and this leads to 2DGE proteomics. This is the oldest type of proteomic setup used, as exemplified in the first proteomic paper [1]. Although no longer in fashion, this proteomic setup still has a lot to offer [2,3]. In this setup, proteins are digested into peptides to carry out the mass spectrometry based identification/characterization (as first described in Henzel et al. [4]), although complete proteins have sometimes been eluted from 2D gels and analyzed directly in the mass spectrometer [5,6]. In the second setup, i.e., mass spectrometry (MS)-based top-down proteomics [7], quantification and analysis are carried out on complete proteins injected in the mass spectrometer, although a variable extent of proteins separation can be applied before injection in the mass spectrometer to deconvolute the samples [8].

In contrast to top-down proteomics, bottom-up proteomics separates, quantifies, and analyzes not complete proteins but peptides produced by the digestion of the proteins. Peptides are analyzed by mass spectrometry, and the resulting data is compared with a database using automated search programs followed by an in silico reassembly of the respective proteins. Intellectually speaking, the method derives from the observation that univocal identification can be obtained from a few peptides coming from the digestion of a protein [4]. Thus, if digestion peptides can be identified well enough against a genome sequence, and if these digestion peptides can be produced, handled, separated, and analyzed reproducibly, why not use them as a representative proxy for proteins? This approach, first described in 1997 by Yates et al. [9], has met considerable success because peptides are very convenient proxies for proteins. They are easy to produce and to handle, separate well by simple (in principle) chromatography, and fragment well in the mass spectrometer (at least from the tryptic peptides that are most often used). By contrast, proteins follow the “3S rule”: they are Sticky (difficult to extract quantitatively), Soft (difficult to fragment neatly in a mass spectrometer), and Separation-unfriendly (despite what can be achieved by 2DGE). 

Thus, looping back to the city noir metaphor, the bottom-up proteomics lamppost sheds bright light, but what are the keys available under this lamppost? As scientists, we know the power of Murphy’s law, which implies, among many other consequences, that convenience and relevance do not often come hand-in-hand. They are rather two sides of a coin—either you see one, or you see the other. 

In fact, the cardinal and insuperable flaw of bottom-up proteomics is the loss of the connectivity that is present between peptides in a complete protein. Thus, bottom-up proteomics only collects peptide-wide information. As peptides coming from an experimentator-driven digestion have no intrinsic functional role (which complete proteins have), protein-wide information must be reconstructed in silico from the actual peptide-wide information, a process that is much less straightforward than it seems. What are the issues here? Peptides generated from and matching just one single protein (isoform) in the database are called unique. Peptides that cannot be clearly assigned to one only but to several proteins or proteoforms are “shared” peptides. Proteoform is defined as all different molecular forms of a protein product from a single gene including its modifications [10]. Those shared peptides do not enable an unambiguous assignment and thus no unambiguous protein identification. This fact is addressed using a process referred to as “protein inference.” Protein identification (and quantification) is usually obtained with so-called peptide search engines that match experimentally obtained peptide spectra to theoretically derived ones based on the sequences of in silico digested protein sequences from a database. The result is a peptide-spectrum match (PSM). Several spectra may match to only one peptide. If, in turn, one PSM does not unambiguously represent one protein, the result will be a list of proteins grouped into a protein group. Conventionally used data search algorithms and programs are SEQUEST [11], Mascot [12], X!Tandem [13] MaxQuant [14], Proteome Discoverer (Thermo Scientific), or Progenesis QIP (Nonlinear). Up-to now, these software packages can handle the protein inference issue only in parts. Over the years several attempts have been made to address this challenge and additional tools have been developed (e.g. in references [15,16,17,18,19,20]), improving the situation, but the protein inference problem is still not fully solved up to now. To make a clear point here, all search algorithms and programs will process the MS/MS data according to personally defined parameters. The result is a list of proteins or protein groups, but it shall be kept in mind that crucial information about the actual and biologically relevant proteoform might have been lost on the way. 

Overall, this reconstruction process is operated against the genetic template, which means, in turn, that bottom-up proteomics operate at the gene product level, i.e., the theoretical protein sequence predicted from the gene. Thus, the final relevance of bottom-up proteomics will depend on the extent to which the gene product is a good functional proxy for the actual proteins produced in the organism of interest.

### 2.2. How Cells Really Work: Multiple Layers of Regulations

Whatever we may think of it, we live in an intellectual frame dominated by the primacy of the genome, as established in 1941 by Beadle and Tatum [21], where a direct causal link between genes and proteins (enzymes at that time) was established (mRNA was to be discovered two decades later [22]). This primacy of the genome lies on very solid ground. At the organismal level, if the gene is not transcribed and translated (for protein-coding genes, of course), nothing will happen at all. This does not mean that the reverse proposition (i.e., once the gene is transcribed, the die is cast) is true.

From a practical standpoint, we can define transcription *sensu lato* as all the processes going from transcription *stricto sensu* to the release of the final, mature mRNA(s). In an omics perspective, this is a very convenient definition, as this is the operational definition of transcriptomics, which handles and measures mature mRNAs. 

It shall not be forgotten that, as their very name says, mRNAs are just messenger molecules. Once they have been produced, they must be translated into proteins, and the proteins mature to become fully active. From a gene perspective, beyond transcription *sensu lato* lie two additional levels of regulations: translational control and post-translational control. 

Translational control may be described as the poor cousin of regulations. From an energy management standpoint, translational control may look like nonsense. Why spend energy on making mRNAs that are not used? There are some physiological situations where it makes perfect sense, such as gametogenesis/early development, and it is no surprise that translational control has been evidenced and studied in detail in this frame [23,24]. However translational control does exist in somatic cells (e.g., in references [25,26,27,28,29,30,31]). In this case, translational control is the parallel of precautionary expenditures for societies: how much are you prepared to spend just in order to be ready for the difficultly predictable? The fact that translational control occurs in all types of organisms, even in prokaryotes where transcription and translation are coupled [32,33], speaks in favor of the importance of this mechanism. 

Post-translational control has been studied much more extensively in the last few decades, mostly through the prism of post-translational modifications (PTMs) (*stricto sensu* the grafting/ungrafting of chemical moieties to the polypeptide backbone of proteins), although protein cleavage and degradation is also an important part of post-translational control. Literally dozens of different post-translational modifications have been described, and it is fair to say that proteomics has been instrumental to the discovery of many of them. Description of even the most common types of modifications is not in the scope of the present paper, and we would prefer to concentrate on them as a regulatory layer. The problem with PTMs is that their consequences are not univocal. When a gene is transcribed, the associated function will eventually be turned on; when transcription stops, it will eventually fade away. For translational control, the situation is also rather simple. When an RNA is a special type of RNP (ribonucleoparticle), it is translationally silent; when this particle is altered, it becomes translated [34]. This straightforwardness does not hold true for PTMs. For example phosphorylation of a protein can be an activating or a deactivating event [35], and the same holds true for acetylation [36]. Thus, the description of the modification landscape of proteins cannot be directly converted into functional inferences. Moreover, it should be kept in mind that protein function modulation can take place by many other, and even more subtle mechanisms, such as the binding of allosteric regulators of changes in loosely bound prosthetic groups, as in the example of cytosolic aconitase where the loss of a single iron atom in an iron-sulfur cluster triggers an activity change from aconitase to RNA-binding [37]. 

When trying to figure out the relevance of the various omics in deciphering changes in cell functioning, the question turns into weighing these different layers of regulations in the cellular life. We believe it is fair to confess that today, we cannot do such a comparison. Once again, we would like to use a metaphor to represent the authors’ views, and this time, it will be a pastry metaphor. With eggs, flour, sugar, butter, baking powder, and water, you can make many different pastries: pies, cakes, muffins, sponge cakes, puff pastries, etc. The only differences lie in the proportions and handling of the ingredients. In our view, transcriptomics is the shopping list, bottom-up proteomics the actual ingredients lists, and top-down proteomics the actual recipe. 

## 3. Position of Bottom-up Proteomics

### 3.1. The Lost Battle of Proteomics: Comprehensiveness

In the wake of the success of the genomic projects, all functional omics have dreamt of comprehensiveness. Because we live in this genetic-primacy world, comprehensiveness shall be understood as the fraction of the genome detectable and measurable by the functional omics, and this view is shared for at least transcriptomics and proteomics, where direct inferences and predictions can be made from the genome. With the development of RNA sequencing, it is fair to say that transcriptomics has reached comprehensiveness [38], while proteomics cannot claim this status, even after more than a decade of extensive effort. There are a series of reasons explaining this situation. 

The first reason lies in the chemical diversity issue. Chemically speaking, mRNAs are always and only a peculiar type of poly(sugar-phosphate), whatever protein they encode; thus, “one size fits all” for the chemical handling of RNA molecules in RNA sequencing. By contrast, proteins come with very different sizes, isoelectric points, hydrophobicities, foldings, etc., so that there is no universal method to handle all of them. This aspect is particularly severe for top-down proteomics, where complete proteins are to be analyzed, but much less for bottom-up proteomics, where peptides have a much lower chemical diversity. Moreover, one protein produces several peptides, so that it can always be reasonably hoped that some of these peptides will behave nicely in the proteomic analysis and thus act as a proxy for the protein. This chemical diversity also makes it difficult to develop universally applicable analytical methods for proteomics corresponding to the sequencing methods in the nucleic acid world.

The second reason lies in the dynamic range. In a mammalian cell, the dynamic expression range of mRNAs spans four orders of magnitude [39], while the protein dynamic expression range of mRNAs spans six orders of magnitude. When it comes to analyzing the needle and the haystack at the same time, these additional orders of magnitude add significant complications.

Another difficulty that shall be mentioned is linked to peptide sharing in closely related proteins that belong to multigene families. In this case, the difficulty comes from the fact that the genetic code is degenerate. Thus, different sequence strings on RNAs can code for completely identical sequence strings at the peptide level, meaning, in turn, that gene products that are easily differentiated at the RNA level may be much more difficult to differentiate at the peptide level. 

Finally, a more practical (but very important) reason lies in the amplification that is possible for nucleic acids, e.g., through Reverse Transcription-Polymerase Chain Reaction (RT)-PCR, which strongly decreases the sensitivity issues, allowing investigations to reach the single cell level [40].

### 3.2. What Room for Bottom-up Proteomics in a Transcriptomic World?

With this obvious and salient issue of comprehensiveness, what is the use of running a proteomic experiment when RNA sequencing can do the job faster and wider? The question is more acute for bottom-up proteomics because this proteomic setup and transcriptomics deliver results at exactly the same level, i.e., the gene product. First of all, there are all the applications where the mRNA level is not relevant or absent, such as body fluids, subcellular proteomics, protein complexes, etc. Then, the question stands at the cellular level (or above) when both transcriptomics and proteomics can be carried out. In this frame, which represents the majority of proteomic papers, the only interest of bottom-up proteomics is to be more relevant, functionally speaking, than transcriptomics. By regression, this question steps back to the correlation between RNA and protein levels. This issue of correlation can be traced to the prehistory of proteomics [41]. With the development of deeper methods, and thus playing with bigger datasets, this question has been the subject of heated debate [39,42,43,44]. The apparent stakes are very simple: if the correlation between RNA and protein levels is high, there is no need to run bottom-up proteomic experiments. If the correlation is low, then it is justified to run proteomic experiments. It seems to us, however, that unless this correlation is the exact scientific question under study, looping back to the question of translational control, this correlation index is not the adequate indicator. In differential proteomics, where the question is to compare two different states by making quantitative comparisons of mRNA or proteins levels, each mRNA can have its own private “translation efficiency factor.” These factors may vary greatly, making a poor overall correlation, but they can be steady when conditions change, so that for each mRNA-protein pair, the change of the mRNA abundance may reflect (or not) the change in protein abundance. In a sense, this question is the first derivative of the correlation question, dealing not with heights but with slopes. The stakes therefore switch to the question of the correlation of the changes in abundances at the mRNA and protein levels. In the literature, there are old and striking examples where the mRNA and protein clocks do not all turn at the same speed, leading, in fact, to opposite changes [45]. The question is to determine whether such discrepancies are rare or frequent. This question has been addressed by Ning et al. [46], and their results are quite clear. In approximately three quarters of the cases, the changes observed for mRNA levels and protein levels went in the same direction. Put more bluntly, this means in turn that one change call out of four made by transcriptomics is completely wrong, amply justifying doing bottom-up proteomics. 

## 4. Escaping from the Transcription Sway: Top-Down Proteomic Returns

The paradigm that is conveyed by transcriptomics and bottom-up proteomics is indeed simple and thus popular: amounts make everything, and their interpretation is direct and simple (see Section 2.2), which is intellectually comfortable. However, a few decades of research on PTMs, not only by proteomics, has shown how important PTM can be in cellular life, going from everyday metabolic control [47] to physiologically or pathophysiologically important decisions mediated by the receptor-kinases signaling cascades (e.g., in Sasi et al. [48]). In this context, it becomes important to really take PTMs into account, not only by mapping the modification sites, but by taking into account how the modifications are integrated on complete proteins creating various proteoforms, which implies in turn to resort to top-down proteomics.

### 4.1. Combinatorial PTMs, A Separation Challenge

The existence of multiple modifications on proteins has been made visible since the earliest days of 2DGE proteomics, as multiple spots of different pI, same molecular weight, and coming from the same protein. As soon as a multiple modified spot can be detected, the question of PTM combination arises. It shall be reminded at this point that spot separation in the isoelectric focusing (IEF) dimension on 2D gels counts only charges. Thus, every modification that induces the same delta in charge will drive the protein at the same place on the 2D gel. As an example, lysine acetylation and serine phosphorylation both result in a −1 charge, and will drive a protein spot at the same place. With the onset of high sensitivity and resolution mass spectrometry, it has become possible to map all modifications that can be present in a single protein spot, and the results can be daunting [49,50]. Calculations made from the theoretical and observed pI render possible the determination of the number of modifications, but any combination of them in the list of detected modifications may occur. This obviously shows that the separation afforded by 2D gels is clearly suboptimal, but even though, the degree of separation possible and the ability to take into account modifications, even without knowing their mapping, can be very valuable, biologically speaking (e.g., in references [51,52,53]). However it is our opinion that many users of 2DGE proteomics do not use fully this ability of the setup, and tend to confuse the part (a change in one form of the protein) for the whole (a change in the total amount of the protein). Because of the importance of PTMs, there is obvious benefit to use a top-down-oriented proteomic setup when possible, either 2DGE based or top-down MS-based. This often highlights interesting PTM-based changes, which are worth validating because they do correspond to cellular regulations, but not at the total protein level. 

### 4.2. Proteisobars, the Ultimate Nightmare in Proteomics

In terms of PTM combination, the resolution afforded in MS-based top-down proteomics goes a step further than the one afforded by 2D gels. For example, a lysine acetylation and a serine phosphorylation are not distinguished in a 2D gel but are in a top-down MS. Despite this improvement, proteomics still faces the wall of proteisobars, i.e., proteoforms that have exactly the same mass, because they bear the exact same combination of modifications, just at different sites. Proteisobars have the same mass (not separable by mass or electrophoresis), the same pI (not separable by IEF unless maybe at extremely high resolution and in a native form [54]), and similar hydrophobicity (not separable on reverse phase chromatography). When introduced simultaneously (because of this lack of separation) in a mass spectrometer and analyzed in a top-down mode, the position of the modifications will be solved but not the relative amounts of the different isobaric variants. 

## 5. “Mehr Licht”: How to Move Proteomic Forward

It is said that “mehr Licht” (more light) were the final words pronounced by the famous German poet and writer Johann Wolfgang von Goethe on his deathbed. Indeed, we need to bring more light to the proteomic lampposts, and especially to those positioned in very dark areas such as top-down proteomics. 

### 5.1. Progress in Bottom-up Proteomics

For bottom-up proteomics, the progresses made on the mass spectrometers themselves have moved the frontier from mass spectrometry to peptide separation. In fact, the glass ceiling of peptide numbers and details in the characterization of the proteins can be moved upward by putting more effort into peptide separation. More separation necessarily means more time spent to gain these details. Today, in addition to time, this improved separation often also means a good deal of craftmanship and dedication, as exemplified by giant peptide separation columns [55], but craftmanship was also needed in the early days of nanoLC ESI MS/MS, which has become a daily routine in many proteomic platforms. Moreover, every additional separation step also holds the risk of introducing higher variability in the analysis workflow. 

Another current trend in bottom-up proteomics is to increase the overall sensitivity to be able to work on very small sample sizes. Once again, this movement parallels the one observed in transcriptomics with single-cell transcriptomics [40]. In this quest, nucleic acids have the tremendous advantage of being amplifiable, which is not true in the case of proteins. In fact, the only PCR that can be experienced in proteomics is Peptide Content Reduction, which is far less positive. Nevertheless, various protocols have been developed to handle very low amounts of proteins (e.g., in references [56,57,58]) in order to face this challenge. In the same trend, the ability to detect proteins at very low levels is still actively investigated, sometimes by means that do not rely on mass spectrometry (e.g., in references [59,60]), and there is no doubt that bottom-up proteomics will perform routinely on a scale close to 1000 mammalian cells in the very near future.

Apart from the analytical developments, there is active research in the numerical and statistical treatment of shotgun proteomics data. In the bottom-up proteomic process, the first issue that arises is that of missing data, which by the way should be decreased if more time was devoted to cleaner peptide separations [55]. This missing data issue has been recognized to be of major importance, and various strategies have been implemented to make “reasonable imputation” of the missing data (e.g., in references [61,62]). The authors of the present paper may just betray their old-school scientist characters by stating that performing data imputation is just an elegant way of creating data from nothing and validating this process ex post by showing that it improves the subsequent statistical analysis. In our opinion, a better (although probably more time and resource consuming) workflow producing less missing data would be highly preferable.

When trying to make sense of proteomic datasets, whichever the proteomic setup used, statistical analyses are mandatory. This need reflects the fuzziness of the data, coming both from the biology and from the proteomic measurements themselves, which prevent us from telling outright the good, the bad, and the ugly. Proteomic data in particular and omic data in general really stretch the power of statistics, as omic experiments are systematically underpowered compared to the richness of the data, making multiple comparisons a cardinal issue. Here again, numerous strategies have been proposed to get the most of proteomic data (e.g., in references [63,64]), and many of them such as the popular volcano plot rely on a combination of fold change threshold and statistical tests results [64]. It should be recalled, however, that a fold change threshold is by definition arbitrary and cannot be applied as such [65]. Just to give an example, shall we implement the same fold change threshold for a very abundant protein that is documented in the shotgun proteomic experiment by more than 10 abundant peptides and for a rare protein that is barely quantified by two low-abundance peptides? Besides the proteomic measurement itself, the cost function for the cell should also be considered. Increasing by 25% the level of a major metabolic protein may represent an awful lot of ATP invested, compared to doubling a rare protein that, in addition, can be easily silenced as needed by a judicious PTM. As we do not know the details of cell functioning yet, it appears safer not to play with fold changes, even if this results in longer lists of modulated proteins at the end [65]. 

Another trend in shotgun proteomic datasets treatment is to use a false discovery rate (FDR) threshold. This FDR is usually set at low values (5% or 1%) [63] in order to limit the number of false positives. It should, however, be kept in mind that, when used at this stage of interpretation of protein abundance changes, FDR in fact represents the likelihood with which a validation experiment based on the protein abundances changes detected by proteomics would fail. As biologists, if we try to figure out the failure rate that we accept or are ready to accept in our targeted biology experiments, it will clearly fall well above 5% and should rather be around 20–30%. As tLangley and Mayr rightly state in [63], there is always a tradeoff between true and false positives, and using a low FDR is, in a sense, accepting the throwing out of some babies with the bathwater. 

In a nutshell, we believe that we shall not forget that, in our applications, statistical analysis should be viewed as nothing other than risk management for the validation experiments that should (or rather must) follow. Thus, thresholds and FDR should be used (or not) and tuned according to this perspective, and not only to reduce the size of the “positive list” coming out of a proteomic experiment. 

It shall not be forgotten, however, that on top of the analytical glass ceiling, there is still the steel ceiling induced by the loss of connectivity between peptides in bottom-up proteomics, which will be an insuperable limitation when details of the cellular regulations are to be investigated. The two functional omic techniques that offer the highest numbers of genes covered are transcriptomics and bottom-up proteomics, but both operate at the gene level products, and therefore do not take into account a large part of cellular regulations. It is impressive to see how this point is neglected, which represents a very good example of cognitive availability bias. What is now presented in recent papers as “progress in proteomics” (and is in fact progress in bottom-up proteomics) only focuses on sensitivity and number issues (e.g., in references [59,60,66]) and thus only on protein identification and never on protein characterization, while this would be the only way to integrate the cellular regulations that are currently not taken into account.

### 5.2. Progress in 2DGE Proteomics: Do and Dare

In our opinion, 2DGE proteomics shall be viewed as a mature, efficient, and cost-efficient way to enter the top-down proteomic field, despite the known limitations of 2D gels for the analysis of hydrophobic [67,68], high molecular weight proteins, and proteins with extreme isoelectric points [69]. Although it is quite clear that the resolving power of 2DGE is insufficient compared to the diversity of proteoforms [49,70], 2DGE is an efficient tool for at least a pre-separation of isoforms. At the separation level, although it is true that narrow gradients can afford a tremendous resolution [54], their systematic use would be extremely resources-consuming and is therefore not widespread, to say the least. Furthermore, the high resolution of narrow pH gradients can deploy only if the proteins are soluble enough around their pI. Otherwise, isoelectric precipitation induces streaking that outweighs the theoretical increase in resolution. Nevertheless, relatively rare proteins can be analyzed by 2DGE proteomics, provided that adequate prefractionation and narrow pH gradients are used [71,72].

However, it seems to us [69] and to others (e.g., in Zhan et al. [70]) that the coupling of high-sensitivity mass spectrometry with 2DGE has a lot to offer in proteomics at the proteoform level. Indeed, high-sensitivity mass spectrometry offers a wealth of peptide information for each 2D spot analyzed. This information can be handled from two different perspectives.

The first perspective focuses on the fact that one spot can contain many different proteins [73]. Evaluating the real proportions of the different proteins present in a single spot from the mass spectrometry data is not as straightforward as it may seem, as the indices used to do so are questionable [42]. Furthermore, going in that direction when performing differential proteomics may be a risky business. It should be kept in mind that proteins do not have the same exact positions from one 2D gel to another. To compensate for these migration changes, we use the staining pattern as a guide to recognize the same spot across a series of gels, and compensate for the migration changes. If we claim to analyze even the minor components of a 2D spot in a comparative proteomic experiment, we make the implicit assumption that the migration alterations of these minor components will follow exactly the migration alterations of the major components, which we detect through the staining. The authors believe that this is a rather risky bet. 

The second perspective focuses on the fact that one spot contains many different modified peptides for the same protein. Up to now, this information has been used mostly to describe the modifications that can take place on a protein without the need to implement prior knowledge of the modifications and using the actual MS/MS spectra to deduce the nature and position of the modifications [49,50]. We believe that this information on modified peptides can be used in differential proteomic experiments to follow the quantitative evolution of the different proteoforms present in a 2D spot between situations of interest. We are aware that this description may lack precision. However, it may be of physiological interest, as exemplified by following cofilin phosphorylations [74]. It should be emphasized that, in this setup, the intensities of identical peptides are compared between spots, so that the peptide inhomogeneity issue present when comparing different peptides between them is absent in this configuration.

Thus, our opinion is that many (if not most) users of 2DGE proteomics do not fully use the power of this proteomic setup, especially for PTMs and in the framework of top-down proteomics. We believe that, when coupled with high sensitivity mass spectrometry, 2DGE proteomics has a lot to offer, and even better at a very reasonable price.

It also seems to us that 2DGE could be more used as a prefractionation tool upstream of MS-based top-down proteomics by eluting complete proteins and injecting them in the mass spectrometer. This is not as technically difficult as it seems, as western blotting has amply demonstrated the possibility of eluting proteins of any size out of a SDS gel (e.g., in Fritz et al. [75]). In the times of Edman sequencing, where the sensitivities were very far from what they are now, scientists have developed methods enabling to concentrate proteins from several 2D spots [76]. However, there is still a strong issue with these techniques, which is linked to the presence of sodium dodecyl sulfate (SDS). SDS is indispensable for the solubilization of proteins and for high resolution separations, and there is indeed very limited flexibility in terms of detergent choice to maintain solubilization and separation [77]. This is why SDS is so widely used for protein elution from 2D gel spots, either on a solid [76] or liquid [5,78] support, or in so called 3D electrophoresis [79,80]. However, SDS must be removed prior to introduction in the mass spectrometer and/or the chromatographic column [81]. This process is plagued by losses for the simple reason that proteins dissolved in SDS are denatured and show a high propensity to precipitate as soon as the SDS is removed, unless another solubilizer of denatured proteins is present, such as urea [82]. Thus, urea-based electrophoresis and especially acid urea electrophoresis (e.g., in Kitta et al. [83]) may be of interest as an interface between classical 2D gels and a top-down mass spectrometry setup, as an acidic pH will strongly limit protein carbamylation. It should also be kept in mind that the SDS electrophoresis process itself may induce oxidative protein modifications, although this has not been described to date in the literature [6]. However some precautions may be worth taking [84], including the replacement of persulfate-based initiator systems by photopolymerization systems [84,85]. 

### 5.3. Progress in MS-Based Top-Down Proteomics: Is Virtue in the Middle?

For this proteomic setup, this paper will focus on the separation challenges upstream of the top-down mass spectrometry itself and not on the MS data production and interpretation. More very valuable information on this can be found in references [86,87,88,89,90]. Indeed, for maximizing the number of proteins that can be analyzed by MS-based top-down proteomics, some protein separation must be applied upfront. As proteins are more efficiently separated by electrophoresis than by chromatography [91], this separation is performed by a low resolution variant of 2D electrophoresis [8], which still poses the problem of SDS removal [81]. Thus, it might be of interest to reverse the order of the separations and start by the SDS electrophoresis separation, as described in rare variants of 2DGE [92,93]. For an easy interface with the Liquid Chromatography-Mass Spectrometry (LC/MS) system, a good setup for the second dimension IEF separation could be to use the so-called offgel separation [94], where proteins are collected directly into a liquid phase. 

Although this may improve both the quantitative yield and the number of proteins amenable to analysis, the proteisobars issue will remain unaddressed. On the one hand, the complete protein cannot be separated from each other, and on the other hand, cutting them into small pieces for bottom-up analyses will make the reconstruction of the proteoforms impossible. So the only remaining solution is the so-called middle-down approaches [95], where proteins are cut into polypeptides in the 5–30 kDa range, which are then separated and analyzed by tandem mass spectromery (MS/MS). The hope is that, by knowing the mass of the total protein, and thus its number and types of modifications, the different chunks of proteins analyzed by the middle-down approaches will allow the user to differentiate the various proteisobars. Ideally, at least two overlapping sets of protein chunks should be produced and analyzed, so that the complete proteins can be reconstructed, exactly as people were sequencing proteins in the first days of Edman sequencing, i.e., before the age of DNA sequencing. 

The difficulty resides indeed in the production of the protein fragments of the adequate size. Ideally, this would mean a protease cutting every 100 amino acids on average, i.e., a protease tightly recognizing a pair of aminoacids. Although many proteases remain to be discovered, this type of cutting does not seem to be widespread. More widespread are “digestive” proteases that cleave after one (or one class) of amino acids, such as trypsin, chymotrypsin, Lys-C, Lys-N, or the same, or specific proteases that recognize a larger site (e.g., thrombin, TEV protease) but thus do not cleave frequently enough for middle-down proteomics. Furthermore, the ideal protease should be able to work in (at least moderately) denaturing environments, as proteomics usually works with generic protocols and thus on denatured proteins. There have been efforts to find such a protease [96], but they have not yet ended in a practical protocol. Thus, one possibility could be to resort to even older methods such as limited proteolysis [97], in which the protein of interest is exposed for a short time to a low concentration of a “digestive” protease and the reaction is stopped by the electrophoretic separation of the starting protein, the fragments, and the proteases. Although the method is generic and uses reagents and separations that are commonplace in proteomics, it requires optimization for every protein to be analyzed, so that it is likely that this approach will be kept as a last resort method. 

## 6. As a Conclusion: Is Proteomics Enlightening?

In this very short conclusive section, we would like to close the parenthesis that we opened at the beginning of this paper. It seems to us that progress in proteomics has been largely driven by the steady and spectacular improvements in the performances of mass spectrometers. This was accompanied by an increasing popularity of counting identifiable and quantifiable peptides and proteins without keeping the reality of the biological regulations in mind. However, we get the impression that it will no longer be the case, and that the bottleneck will swing back to the separation prior to the mass spectrometry, i.e., from science in the vacuum to science in water. Beside the avenues for technical improvement that we have just touched in the previous section, we strongly believe that the key issue, as we mentioned in the introduction of this paper, is to interpret the omic data (and in our case the proteomic data) at exactly the right level, without intellectual shortcuts that are often true misinterpretations. Only at this price will the proteomic literature deliver on its promises, and the best way to avoid overinterpretations will always bear the name of validation [98].
